# 

**Published:** 1816-01

**Authors:** 


### JOURNAL AND REVIEW.

CONDUCTED BY

S.G.O. INDEXED

WILLIAM SHEARMAN, M. D.
Licentiate of the Royal College of Physicians, and Physician to the London Dispensary;

JAMES JOHNSON, Esq.
Surgeon to His Royal Highness the Duke of Clarence's Household; and Author of "The Influence of Tropical Climates on European Constitutions."

AND

SHIRLEY PALMER, M. D.
Of the University of Glasgow, and Member of the Royal College of Surgeons, London.

VOL. I.

FROM JANUARY TO JUNE, 1816.

SURGEON GEN'L'S OFFICE LIBRARY. 63384

*LONDON.*
PRINTED FOR THE PROPRIETORS,
BY WILLIAM THORNE, RED LION COURT, FLEET STREET.
Sold by T. and G. UNDERWOOD, No. 32, Fleet Street; HIGHLEY and SON, 174, Fleet Street; J. CALLOW, Crown Court, Princes Street, Soho; A. BLACK, Edinburgh; and GILBERT and HODGES, Dublin.

### CORRESPONDENCE.

The Editors have to acknowledge the communication of Mr. Pym, relative to the Gibraltar fever, which was too late for the *Original Department* of the Journal, but shall positively appear in their next Number. They have in some measure atoned for this delay, by subjecting the manuscript to the perusal of some eminent medical characters, who are preparing reports to Government on Mr. Pym's work, and have thus given Mr. Pym every advantage in their power.

There never was perhaps a more momentous question submitted to the Medical world, than that which Government is now agitating relative to the fever alluded to; and therefore the Editors will give every facility to Mr Pym, and to every other medical man interested or experienced in the question under dispute, in order that TRUTH may be established, which must be the object of both parties, as well as of the Editors.

The Editors call, in the name of humanity, on every naval and military practitioner, who has seen the fevers of the East and West Indies, or those of Cadiz, Gibraltar, and other parts of the Mediterranean, for candid and impartial statements relative to the following points. 1st. Whether that concentrated yellow fever,* which runs its course (whether fatal or otherwise) in a few days, generally from three to five, has ever been known to

---

* Vide description of this fever in Mr. Pym's work, or New Med. and Phys. Journal. Also in the section on Yellow fever, in Mr. Johnson's "Influence of Tropical Climates."

affect the human constitution more than once. 2nd. Whether *relapses* are usual in the same. 3rd. Whether or not it has appeared *contagious.* The question is now a national one, involving the dearest interests of society, and consequently demanding a full, a slow, and a most minute investigation. Every piece of information calculated to throw light on the above topics will be thankfully received by the Editors.

Mr. H. Bailey's case of contracted Intestine (with a drawing); and Mr. Howship's case of diseased Omentum, will appear in our next.

A great variety of interesting articles, intended for our Miscellaneous Department, are unavoidably deferred for want of room.

---

### *Diseases and Casualties in London in the Year* 1815.

| | |
|---|---|
| Abortive and Stillborn | 804 |
| Abscess | 105 |
| Aged | 1757 |
| Ague | 5 |
| Apoplexy & Suddenly | 421 |
| Aſthma | 680 |
| Bedridden | 2 |
| Bile | 5 |
| Bleeding | 23 |
| Burſten and Rupture | 34 |
| Cancer | 88 |
| Chicken Pox | 2 |
| Childbed | 232 |
| Colds | 16 |
| Colic, Gripes, &c. | 26 |
| Conſumption | 4210 |
| Convulſions | 3324 |
| Cough, and Hooping Cough | 729 |
| Cramp | 4 |
| Croup | 87 |
| Diabetes | 6 |
| Dropſy | 792 |
| Epilepſy | 1 |
| Evil | 7 |
| Fevers of all kinds | 1309 |
| Fiſtula | 3 |
| Flux | 65 |
| French Pox | 22 |
| Gout | 67 |

| | |
|---|---|
| Gravel, Stone, and Strangury | 16 |
| Grief | 5 |
| Headmoldſhot, Horſeſhoe head, and water in the head | 383 |
| Impoſthume | 4 |
| Inflammation | 953 |
| Influenza | 1 |
| Jaundice | 90 |
| Lethargy | 1 |
| Livergrown | 46 |
| Lumbago | 3 |
| Lunatick | 228 |
| Meaſles | 711 |
| Miſcarriage | 2 |
| Mortification | 306 |
| Palpitation of the Heart | 6 |
| Palſy | 163 |
| Pleuriſy | 18 |
| Quinſy | 5 |
| Raſh | 1 |
| Rheumatiſm | 9 |
| Scrophula | 3 |
| Scurvy | 4 |
| Small Pox | 725 |
| Sore Throat | 5 |
| Sores and Ulcers | 11 |
| Spaſm | 36 |
| St. Anthony's Fire | 9 |

| | |
|---|---|
| Stoppage in the Stom. | 23 |
| Surfeit | 1 |
| St. Vitus's Dance | 2 |
| Teeth | 447 |
| Thruſh | 118 |
| Tumor | 3 |
| Water in the Cheſt | 30 |
| Worms | 3 |
| Bit by Dogs | 2 |
| Broken Limbs | 1 |
| Bruiſed | 2 |
| Burnt | 32 |
| Drowned | 132 |
| Exceſſive Drinking | 8 |
| Executed | 8 |
| Found Dead | 25 |
| Fractured | 2 |
| Frighted | 5 |
| Killed by Falls and ſeveral other Accidents | 76 |
| Killed themſelves | 47 |
| Murdered | 1 |
| Overlaid | 1 |
| Poiſoned | 1 |
| Scalded | 10 |
| Shot | 10 |
| Suffocated | 9 |
| Total | 363 |

| | | | |
|---|---|---|---|
| Chriſtened | Males | 12281 | In all 23414 |
| | Females | 11133 | |
| Buried | Males | 9882 | In all 19560 |
| | Females | 9678 | |

Whereof have died,

| | | | |
|---|---|---|---|
| Under Two Years of Age | 5200 | Sixty and Seventy | 1621 |
| Between Two and Five | 1916 | Seventy and Eighty | 1221 |
| Five and Ten | 870 | Eighty and Ninety | 674 |
| Ten and Twenty | 677 | Ninety and a Hundred | 167 |
| Twenty and Thirty | 1425 | A Hundred | 2 |
| Thirty and Forty | 1824 | A Hundred and One | 1 |
| Forty and Fifty | 2075 | A Hundred and Three | 1 |
| Fifty and Sixty | 1886 | | |

Decreaſed in the Burials this Year 223.

